# Brainstem neuropathology in two cases of COVID-19: SARS-CoV-2 trafficking between brain and lung

**DOI:** 10.1007/s00415-021-10604-8

**Published:** 2021-05-18

**Authors:** Gaetano Bulfamante, Tommaso Bocci, Monica Falleni, Laura Campiglio, Silvia Coppola, Delfina Tosi, Davide Chiumello, Alberto Priori

**Affiliations:** 1 Neurology Unit I, Department of Health Sciences, University of Milan, Via Antonio di Rudinì 8, 20142 Milan, Italy; 2 Pathology and Medical Genetics Unit, University of Milan, Milan, Italy; 3 Intensive Care, Anesthesia and Resuscitation Unit, University of Milan, Milan, Italy; 4 ASST Santi Paolo & Carlo and Department of Health Sciences, University of Milan, Milan, Italy; 5 ``Aldo Ravelli’’ Center for Neurotechnology and Experimental Brain Therapeutics, University of Milan, Milan, Italy

**Keywords:** COVID-19, SARS-CoV-2, Brainstem, Neuropathology, Medulla oblongata, Neurological COVID-19

## Abstract

**Introduction:**

SARS-CoV-2 might spread through the nervous system, reaching respiratory centers in the brainstem. Because we recently reported neurophysiological brainstem reflex abnormalities in COVID-19 patients, we here neuropathologically assessed structural brainstem damage in two COVID-19 patients.

**Materials and methods:**

We assessed neuropathological features in two patients who died of COVID-19 and in two COVID-19 negative patients as controls. Neuronal damage and *corpora amylacea* (CA) numbers /mm^2^ were histopathologically assessed. Other features studied were the immunohistochemical expression of the SARS-CoV-2 nucleoprotein (NP) and the Iba-1 antigen for glial activation.

**Results:**

Autopsies showed normal gross brainstem anatomy. Histopathological examination demonstrated increased neuronal and CA damage in Covid-19 patients’ medulla oblongata. Immunohistochemistry disclosed SARS-CoV-2 NP in brainstem neurons and glial cells, and in cranial nerves. Glial elements also exhibited a widespread increase in Iba-1 expression. Sars-Co-V2 was immunohistochemically detected in the vagus nerve fibers.

**Discussion:**

Neuropathologic evidence showing SARS-CoV-2 in the brainstem and medullary damage in the area of respiratory centers strongly suggests that the pathophysiology of COVID-19-related respiratory failure includes a neurogenic component. Sars-Co-V2 detection in the vagus nerve, argues for viral trafficking between brainstem and lung.

**Supplementary Information:**

The online version contains supplementary material available at 10.1007/s00415-021-10604-8.

Dear Sirs,

Increasing evidence over time has associated SARS-CoV-2 infection with widely ranging neurological complications [6, 14, 15, 18]. Whether SARS-CoV-2 spreads to brainstem respiratory centers, adding a neurogenic component to the respiratory failure, nevertheless remains open to debate [2, 5]. Before SARS-CoV-2 emerged on a global scale, convincing evidence showed that other coronaviruses can invade the brainstem in animals [3, 16] and humans [1, 8] through different ways comprising a prion-like mechanism and hematogenous spread [2, 10, 17]. Accordingly, we have recently reported the clinical and neurophysiological brainstem involvement in patients with severe COVID-19 [4].

To see whether the neurological and neurophysiological findings we previously described depend on structural damage [4], we neuropathologically assessed the brainstem in two patients who died of COVID-19. Both patients, men, aged 56 and 58, with recent hyposmia and hypo/dysgeusia, died of respiratory failure due to COVID-19 related pneumonia. They were hospitalized 2 and 14 days after symptom onset and were intubated one for 18 days (9 days after hospital admission) and the other for 9 days (7 days thereafter); both died about one month after the diagnosis. Nasopharyngeal swabs remained positive from admission to autopsies. In both cases, a further tracheobronchial swab was also obtained immediately before the autopsy.

Neuropathological data were acquired from four autopsies done < 3 h after death: two were from patients who died of COVID-19 and two from COVID-19 negative subjects who died of non-neurological conditions (aged 82 and 84, only one intubated). None of these 4 patients suffered from known underlying neurological diseases or had a history of neurological disorders. The brainstem was formalin-fixed for 48 h as previously described [7]. The fixative was frequently changed thus ensuring tissue antigenicity. Also, the short interval elapsing between death and tissue sampling ensured good tissue quality, with excellent morphology and maintained antigenic properties.

To evaluate neuronal damage we calculated morphologically damaged cell percentages at trigeminal nuclei level in the pons and *medulla oblongata* (MO). A further tissue damage marker assessed was the *corpora amylacea* (CA) number per mm^2^, at the pontine and medullary level. CA are predominantly glial and extracellular glycoprotein inclusions, suggesting astroglial activation [12]. SARS-CoV-2 nucleoprotein (NP) expression (Sino Biological; Rabbit MAb; 1:800; detection with magenta) and the glial activation Iba-1 marker (Invitrogen; Rabbit Polyclonal; 1:200; detection in DAB), glial fibrillary acid protein (GFAP) and CD68 antigens, as different markers of astrogliosis and microgliosis, were assessed by immunohistochemistry with an automated immunostainer (DAKO OMNIS), in the same regions where the neuronal damage and CA were evaluated. Antibodies against CD3, CD4 and CD8 were finally used to detect T lymphocyte inflammatory infiltration.

The two COVID-19 patients had macroscopically normal brainstems (Fig. 1). Microscopy in both cases showed more damaged neurons in the *medulla* than in the pons (*MO, reticular formation* = case 1: 43.3%, case 2: 57.7% *vs Pons, reticular formation* = case 1: 16.1%, case 2: 16.5%. *MO, spinal trigeminal nucleus* = case 1: 48%, case 2: 45.4% *vs Pons, trigeminal motor nucleus* = case 1: 9.7%, Case 2: 22.0%). The number of damaged neurons in the MO was higher in COVID-19 positive than in COVID-19 negative patients (*MO, reticular formation* = control 1: 3.27%, control 2: 6.84%). The number of *CA per* mm^2^, was higher in the *medulla* than in the pons (*MO, reticular formation* = case 1: 14.09/mm^2^, case 2: 9.65/mm^2^ *vs Pons, reticular formation* = 10. 9/mm^2^, case 2: 7.43/mm^2^) and in COVID-19 patients than in controls (*MO, reticular formation* = control 1:1.12/mm^2^, control 2: 7.44/mm^2^) (Fig. 2.I). Unlike COVID-19 negative patients, COVID-19 positive patients had a marked increase in activated glial elements immunoreactive to Iba-1, despite the younger age than controls (Supplementary Fig. 1); in particular, GFAP expressing cells increased slightly, concomitant to unvaried CD68 expression in glial cells (as expected in inflammatory, but not degenerative, diseases of the nervous system), but strongly increased Iba-1 immunoreactivity in the microglial compartment. NP immunoreactivity demonstrated SARS-CoV-2 virus in brainstem neurons and glial cells, and occasionally in pontine and medullary parenchymal vessel endothelium (Fig. 2.II). The vagus nerve fibers emerging from the MO surface showed intense NP immunostaining (Fig. 2.II, sections d and e). T lymphocyte infiltration also increased, with perivascular localization, as expected in a viral infection.

**Fig. 1:**
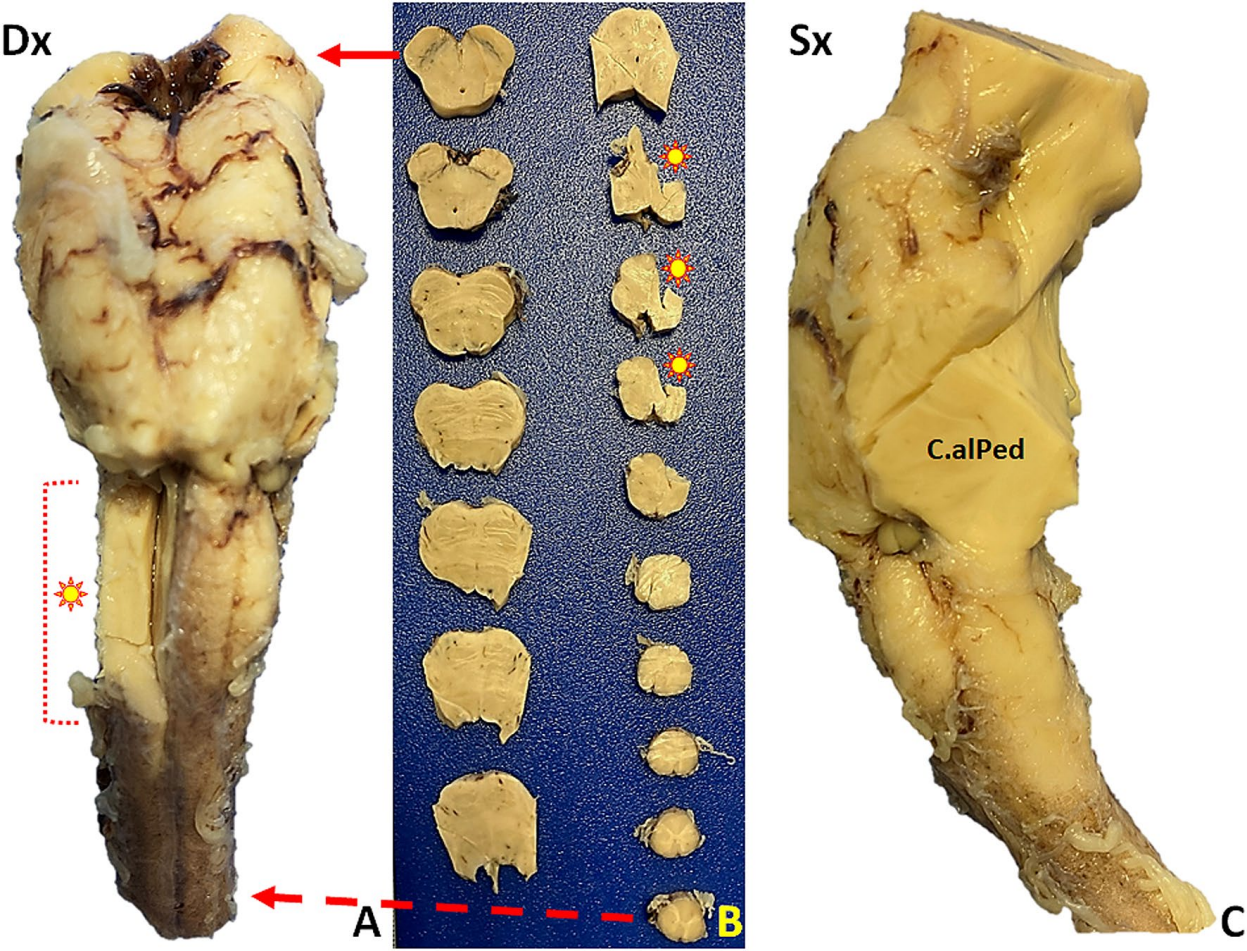
Macroscopic brainstem appearance in COVID-19-patients. **A **Anterior surface after major arterial vessel removal: no evidence of pathological changes. Dx: right side. Asterisk: right *medulla oblongata* (bulb) area removed for tissue sampling. **B** Transverse brainstem sections. No evidence of gross pathological changes. The solid arrow (top) indicates the section at cerebral peduncle and the *substantia nigra* levels. The asterisks correspond to the sampled area in image **A**. The dashed arrow (bottom) corresponds to the most caudal *medulla oblongata* section. **C** Left brainstem (dorsal face on the right). C.alPed: cerebellar stalk

**Fig. 2:**
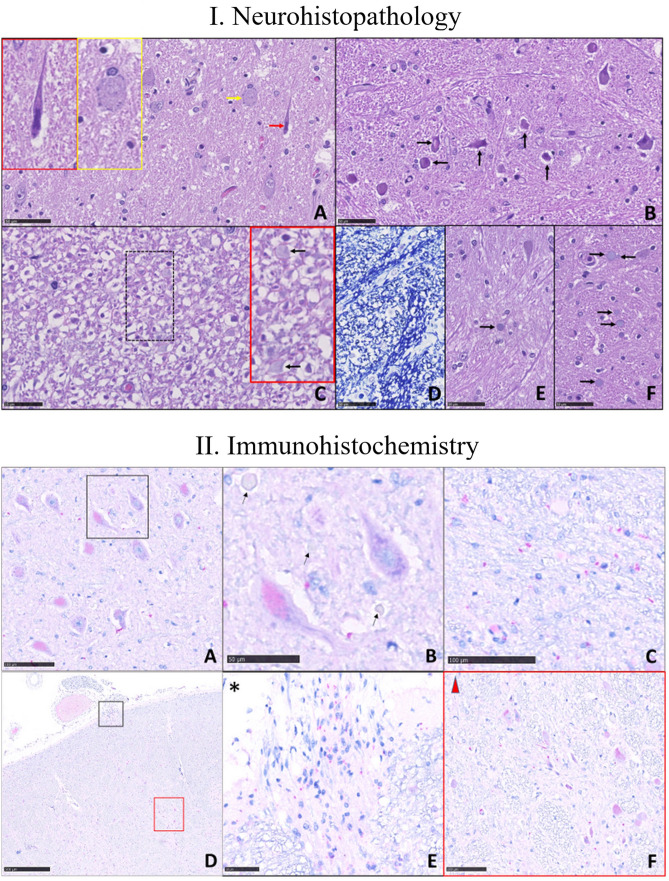
Microscopic brainstem examination. I. Images are obtained by Nanozoomer Hamamatsu s360 and magnification is represented as scale bare. Histopathological features of nervous tissue damage in the pons and *medulla oblongata* (MO; scale bare: 50 µm). **A**–**D** and **F** are from COVID-19 patients. **A** Pons, V motor nucleus. Different types of neuronal damage: neurons with condensed chromatin and shrunken cell body (black arrow), and neuronal chromatolysis (yellow arrows), magnified respectively in the black and yellow insert at the top left. Tissue oedema is absent (haematoxylin & eosin [H&E] staining). **B** MO, reticular formation: the neurons (some indicated by arrows) are damaged. Tissue oedema is absent (H&E). **C** MO, reticular formation: axonal damage; the myelin sheaths are detached from the axons and fibers vary in diameter; some *corpora amylacea* are visible (arrows) in the red insert (magnification of the hatched area; H&E). **D** MO, reticular formation: myelin sheaths (in dark blue) vary in thickness and size (Klüver-Barrera staining). **E** Patient unaffected by COVID-19 (control). Pons, reticular formation. The parenchyma appears well preserved and contains a single *corpus amylacea* (arrow; H&E). **F** Pons, reticular formation. The parenchyma in this patient with COVID-19 appears characterized by many *corpora amylacea* (arrows) (H&E). II. Immunohistochemistry. Nucleoprotein (NP) neuronal immunoreactivity (in magenta) in the medulla oblongata (MO) and pons. **A** MO. A low-power view of NP positive (intracytoplasmic magenta staining) and negative neurons in the motor nucleus of the trigeminal nerve (scale bare: 100 µm). **B** Magnification of two neurons, one infected by the virus (lower left) and one negative, with basophilic tigroid substance granules; some *corpora amylacea* are seen (arrows; scale bare: 50 µm). **C** Red granules show viral NP immunoreactivity in glial elements surrounding neurons in the motor nucleus of the trigeminal nerve (scale bare: 100 µm). **D** Low magnification of a bulbar section at the level of vagus nerve fibers (scale bare: 500 µm). **E** and **F **NP immunoreactivity is visible in nerve fibers entering the brainstem (**E**, black inset with asterisk; scale bare: 50 µm) and in neurons of the nucleus ambiguous (**F**, red insert with triangle; scale bare: 100 µm)

Our findings show SARS-CoV-2-related brainstem involvement. In our cases (Fig. 2.I.a–b), the absence of tissue edema, red cell changes, karyolysis and pyknosis*,* all histopathological features indicating hypoxemia, argue against—though do not completely exclude—pathological findings arising from hypoxic damage. The immunohistochemical evidence showing Sars-CoV-2 in the brainstem and astroglial activation (increased CA and Iba-1 expression) agrees with recent data [13]. For example, Matschke and co-workers showed a preferential lower medulla involvement and SARS-CoV-2 NP intraneuronal NP localization [13]. In two other patients with COVID-19-related brainstem encephalitis without viral RNA detection in the brain (the interval between death and autopsy was 9 days), although the investigators concluded that their findings are para-infectious phenomena related to the systemic pro-inflammatory and hypercoagulable conditions, they neuropathologically observed vascular injury and thrombotic microangiopathy in the brainstem [11]. Our current observations distinctly differ from previous findings mainly owing to the autopsy protocol we applied (< 3-h elapsing between death and autopsies), preventing autolytic post-mortem phenomena and thus maximally preserving tissues [7]: for instance, Matschke and co-workers did autopsies from 1 to 9 days after death (mean 3.3 days) [13]. Also, unlike Jensen and colleagues [11], we assessed astroglial activation by testing Iba-1 immunoreactivity [11].

Most importantly, Sars-Co-V2 immunohistochemically detected in the vagus nerve fibers is an original and unreported finding, suggesting viral trafficking between brainstem and lung. Whether the virus moves from the brain to the lung, or vice versa remains unknown.

Overall, brainstem neuropathological involvement agrees with neurophysiological brainstem abnormalities [4] and neuroradiological evidence describing rhombencephalitis in COVID-19 [9, 19]. Clinical, neuropathological, neurophysiological and neuroradiological data therefore indicate that severe COVID-19 involving the central nervous system targets the brainstem. Neuropathologic evidence showing SARS-CoV-2 in the brainstem and medullary damage in the respiratory center areas strongly suggests that the pathophysiology of COVID-19 related respiratory failure includes a neurogenic component.

## Supplementary Information

Below is the link to the electronic supplementary material.

Supplementary file1 (TIF 3133 kb)

## Data Availability

The corresponding author has full access to data and has the right to publish such data. Data will be available upon reasonable request to the corresponding author.
